# Abdominal drainage versus no drainage after distal pancreatectomy: study protocol for a randomized controlled trial

**DOI:** 10.1186/s13063-019-3442-0

**Published:** 2019-06-07

**Authors:** Joerg Kaiser, Willem Niesen, Pascal Probst, Thomas Bruckner, Colette Doerr-Harim, Oliver Strobel, Phillip Knebel, Markus K. Diener, André L. Mihaljevic, Markus W. Büchler, Thilo Hackert

**Affiliations:** 1 Department of General, Visceral and Transplantation Surgery, University Hospital Heidelberg, Im Neuenheimer Feld 110, 69120 Heidelberg, Germany; 2 Institute of Medical Biometry and Informatics, University of Heidelberg, Im Neuenheimer Feld 130.3, 69120 Heidelberg, Germany

**Keywords:** Distal pancreatectomy, Drain, Postoperative complications, Pancreatic fistula

## Abstract

**Background:**

The placement of prophylactic intra-abdominal drains has been common practice in abdominal operations including pancreatic surgery. The PANDRA trial showed that the omission of drains following pancreatic head resection was non-inferior to intra-abdominal drainage in terms of postoperative reinterventions and superior in terms of clinically relevant pancreatic fistula rate and fistula-associated complications. The aim of the present PANDRA II trial is to evaluate the clinical outcome with versus without prophylactic drain placement after distal pancreatectomy.

**Methods:**

The PANDRA II trial is a mono-center, randomized controlled, non-inferiority trial with two parallel study groups. In the control group at least one passive intra-abdominal drain is placed at the pancreatic resection margin. In the experimental group no drains are placed. The primary endpoint of this trial will be the Comprehensive Complication Index (CCI) measuring all postoperative complications within 90 days. Secondary endpoints are in-hospital mortality and morbidity, including the rates of postoperative pancreatic fistula, chyle leak, postpancreatectomy hemorrhage, delayed gastric emptying, reinterventions and reoperations, surgical site infection, and abdominal fascia dehiscence. Moreover, length of hospital stay, duration of intensive care unit stay, and the rate of readmission after discharge from hospital (up to day 90 after surgery) are assessed. We will need to analyze 252 patients to test the hypothesis that no drainage is non-inferior to drain placement in terms of the CCI (δ 7.5 points) in a one-sided *t* test with a one-sided level of significance of 2.5% and a power of 80%.

**Discussion:**

The results of the PANDRA II trial will help to evaluate the effect of an omission of prophylactic intraperitoneal drainage on the rate of complications after open or minimally invasive distal pancreatectomy.

**Trial registration:**

German Clinical Trials Register (DRKS), DRKS00013763. Registered on 6 March 2018.

**Electronic supplementary material:**

The online version of this article (10.1186/s13063-019-3442-0) contains supplementary material, which is available to authorized users.

## Background

The placement of prophylactic intra-abdominal drains to reduce postoperative surgical complications has been common practice in abdominal surgery for decades. Recent studies, however, have failed to show any benefit of routine drainage following many abdominal resections including hepatobiliary [1, 2], gastric [3, 4], and colorectal surgery [5, 6]. These studies have shown that surgery can be performed safely without prophylactic drainage. Although the use of prophylactic drains was shown to be unnecessary in other resections, many surgeons fear that abandoning prophylactic drainage after a pancreatic resection could be detrimental due to the high rate and severe consequences of pancreatic fistula. While mortality after pancreatic resections has been significantly reduced, postoperative morbidity following pancreatic surgery remains a concern and is as high as 40–50% even in specialized centers [7–9]. For many surgeons the rationale for inserting drains following pancreatic surgery is to evacuate the blood, bile, pancreatic juice, or chyle that may accumulate after pancreatic surgery, which can serve as an early warning sign of postoperative pancreatic fistula and its associated complications such as postpancreatic hemorrhage. However, the majority of patients do not develop postoperative pancreatic fistula, and experiences with drains in other operations [1–6] suggest that unnecessary drains can cause complications. Drains might serve as a port of entry for bacteria and turn non-infected postoperative fluid collections into abscesses and infected fistula. In addition, drains might cause tissue trauma from suction, erode anastomoses, and cause intestinal leaks [10]. Several randomized and non-randomized trials have attempted to identify risk constellations where placement of abdominal drains after pancreatic surgery seems to be justified [7, 10–13]. Other studies have tried to explore the effectiveness of early versus late removal of abdominal drains [14, 15]. In 2017, the PANDRA trial showed that the omission of drains following pancreatic head resection was non-inferior to intra-abdominal drainage in terms of postoperative reinterventions and superior in terms of clinically relevant pancreatic fistula rate and fistula-associated complications [16]. Therefore, some surgeons have abandoned the routine use of drains in pancreatic surgery. Nonetheless, most pancreatic surgeons continue to use a prophylactic drain. Up to now it is unclear whether routine abdominal drainage after distal pancreatectomy has any effect on the reduction of postoperative complications. The aim of the present PANDRA II trial is to evaluate the clinical outcome of prophylactic drain placement versus no drainage after open or minimally invasive distal pancreatectomy with respect to postoperative complications.

## Study design

### Objectives and hypotheses

The PANDRA II trial investigates differences in the postoperative course after distal pancreatectomy comparing the surgical technique with placement of an abdominal drain versus no placement of an abdominal drain after open or minimally invasive distal pancreatectomy.

The following hypotheses will be tested:

- H0: The risk of developing postoperative complications is different between the two groups.
- H1: The risk of developing postoperative complications is equal in both groups.

### Study registration, ethics, and consent

Before inclusion of the first patient, the trial protocol was approved by the ethics committee of the University of Heidelberg (Ethikkommission Medizinische Fakultät Heidelberg, S-675/2017) and the trial was registered (DRKS00013763; UTN: U1111-1207-3031). The trial will be conducted at the Clinical Trial Center (KSC) of the Department of General, Visceral and Transplantation Surgery, University Hospital Heidelberg in the context of Good Clinical Practice and in accordance with the Declaration of Helsinki. Randomization and data management are performed by the KSC. The statistical analysis will be performed independently by the Institute of Medical Biometry and Informatics (IMBI) of the University of Heidelberg. The PANDRA II trial is designed as a mono-center, randomized controlled, non-inferiority trial with two parallel study groups. All patient-related information is subject to medical confidentiality and to medical secrecy according to the European General Data Protection Regulation (DSGVO — Datenschutzgrundverordnung), the Federal Data Protection Act (Bundesdatenschutzgesetz), and the State Data Protection Act (Landesdatenschutzgesetz). Third parties will not have any insight into original data.

### Study population

All patients assigned for distal pancreatectomy at the Department of General, Visceral and Transplantation Surgery, University of Heidelberg, will be screened for eligibility before the operation. Eligible for participation are all patients planned for open or minimally invasive distal pancreatectomy for pancreatic disease. Patients must be at least 18 years of age and provide written informed consent. Patients have to be able to understand character and individual consequences of the clinical trial. The following exclusion criteria have been defined: (1) indication for pancreatic resection with a pancreaticojejunal anastomosis, (2) American Society of Anesthesiologists (ASA) physical status classification ≥4, (3) impaired mental state or language barriers impeding informed consent, (4) participation in another interventional trial with interference of intervention and outcome of this trial (Table 1). During this preoperative visit, patients will be informed about the clinical problem of postoperative complications, the timeline of the PANDRA II trial, and the possible risks and benefits of participation before they will be asked to give their written informed consent.

**Table 1:** Eligibility criteria

Inclusion criteria	Exclusion criteria
• Surgical indication for open or minimally invasive distal pancreatectomy • Aged 18 years and over • Ability of subject to understand character and individual consequences of the clinical trial • Informed consent provided	• The subject has a surgical indication for pancreatic resection with a pancreaticojejunal anastomosis • American Society of Anesthesiologists (ASA) physical status classification ≥ IV • Impaired mental state or language problems of the subject • Participation in another interventional trial with interference of intervention and outcome of this trial

### Outcome parameters

Overall postoperative morbidity assessed with the Comprehensive Complication Index (CCI) [17] was chosen as primary endpoint since this parameter considers the patient’s perspective as well as parameters of surgical effectiveness. A score of 0 indicates no complications, whereas 100 is equivalent to death. The index is based on the established Clavien-Dindo classification [18]. The score is validated for the pancreatic surgical population, and a difference of 10 is regarded as a clinically relevant difference [17]. An endpoint reflecting the entire spectrum of complications like the CCI is highly appropriate to compare two different surgical strategies for the abdominal cavity. The score will be calculated respecting all complications within 3 months after the index operation.

Secondary endpoints are in-hospital mortality and also postoperative morbidity up to day 90 after the index surgery, including frequency of postoperative pancreatic fistula according to the definition of the International Study Group of Pancreatic Surgery (ISGPS) 2017 [19]; lymphatic fistula according to the definition of the ISGPS 2017 [20]; postoperative bleeding according to the definition of the ISGPS 2007 [21]; reinterventions and reoperations including computed tomography (CT)-guided placement of drains due to intra-abdominal fluid collection, intra-abdominal bleeding, and/or pancreatic and lymphatic fistula; delayed gastric emptying according to the 2007 ISGPS definition [22]; surgical site infection according to Centers for Disease Control and Prevention (CDC) definition; and abdominal fascia dehiscence. Moreover, operation time and length of hospital stay, duration of ICU stay, as well as the rate of readmission after discharge from hospital (up to day 90 after surgery) are assessed.

### Randomization

All participating patients are randomized intraoperatively once the surgical decision to perform a distal pancreatectomy has been made by the attending surgeon. The randomization will be performed prior to pancreatic resection. Block randomization with varying block sizes is performed with a 1:1 allocation ratio. A computer generated random list was created. The allocation is performed by opening sequentially numbered opaque envelopes containing a card marked “Drainage” or “No Drainage”. The randomization process and assignment of the patients to the respective trial intervention are performed by staff members of the KSC at the University Hospital Heidelberg. If the surgical procedure of a distal pancreatectomy is not accomplished after randomization, e.g., due to inoperability or the need for total pancreatectomy, the patient is excluded from final analysis.

### Standardized surgical approach

#### For both groups

Open or minimally invasive distal pancreatectomy is performed by the surgeon according to local practice. Conversion from laparoscopic to open resection will be documented in the case report form, but does not lead to exclusion of a patient. Additional partial resection of larger veins or arteries (e.g., the superior mesenteric/portal vein or celiac trunk) is allowed. Furthermore, patients with additional resection of other organs (e.g., colon, liver, spleen) or even distal pancreatectomy combined with multivisceral resection are also eligible. The decision to perform an additional splenectomy or cholecystectomy is at the surgeon’s discretion. The type of abdominal incision (longitudinal or transverse laparotomy) as well as the placement of trocars in terms of laparoscopic pancreatic resections is determined by the surgeon performing the procedure. After exploration of the abdominal cavity, the pancreas is revealed and transected by scalpel or by stapler as no differences in pancreatic fistula rate have been reported between different transection techniques. A coverage procedure with, e.g., the falciform ligament or suturing the ventral and dorsal surfaces of the gland by single stitches or running suture of the pancreatic remnant is at the discretion of the surgeon performing the procedure and will be documented. Further manipulation of the pancreatic remnant such as the use of fibrin glue or reinforcement with meshes is not allowed, as these methods have failed to show any benefit [23]. The use of somatostatin and its analogs is not standardized in the trial protocol, but it needs to be documented and will be analyzed separately for both groups.

#### Control group

In the control group at least one drain (open-circuit silicone drain or closed-circuit silicone drain) is placed at the pancreatic remnant for percutaneous drainage just before abdominal wall closure. Amylase values are measured on the third postoperative day in the drained fluid. There is no restriction on the number of days the drains should remain. They can be removed whenever they are no longer needed, justified by the clinical state of the patient.

#### Experimental group

In the experimental group no drains are placed.

### Postoperative data collection and blinding

**Fig. 1:**
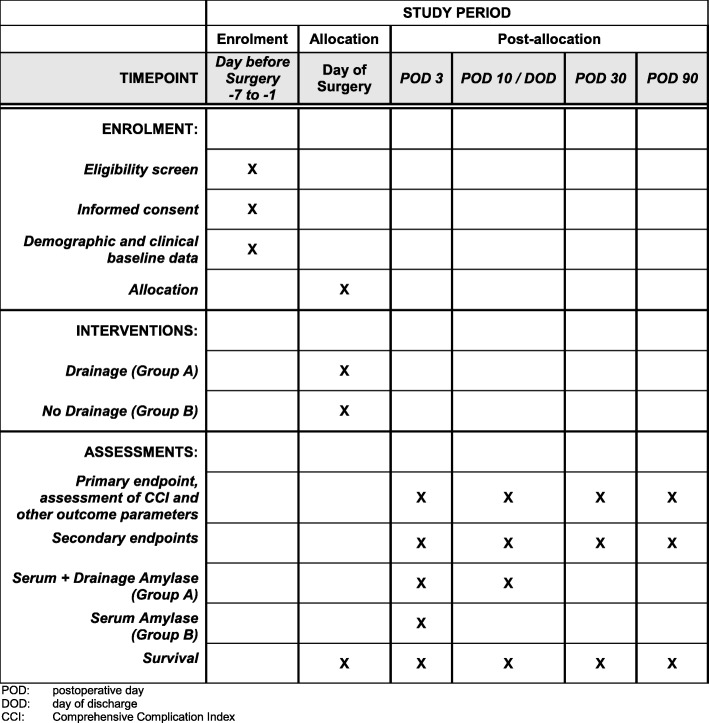
Enrollment, interventions, and assessment schedule of the PANDRA II trial: course of examinations (modified SPIRIT figure)

Postoperative data collection is performed at prespecified time points. Regular visits with study patients on all wards will be performed by clinical investigators and study nurses from the clinical study center in order to collect information on the primary and secondary outcome parameters and to identify any postoperative complication. Two postoperative study visits (3rd postoperative day and 10th postoperative day/ day of discharge) and two follow-up telephone interviews after discharge (30 days and 90 days postoperatively) will be performed (Fig. 1). All surgeons must describe the technique they performed on the pancreas on a standardized form that is sent to them by email on the day of the index operation to record whether patients were treated according to the study protocol and were assigned to their groups truly at random. Blinding to the treatment allocation for participants, research assistants, operating surgeons, data collectors, and outcome assessors is difficult — if not impossible — as drain placement is obvious. However, the primary and secondary outcome measures are objective endpoints that are not influenced by blinding. Therefore, only data analysts will be blinded in this trial.

### Safety aspects

All postoperative complications are monitored during hospital stay and follow-up examinations (up to day 90). Major complications are defined as complications graded IV and V according to the Clavien-Dindo classification [18]. (The Clavien-Dindo classification grades are listed in Additional file 2). The coordinating investigator must be informed about these complications. Additionally, there are evaluations of mortality after including 30 and 90 patients, respectively, into the study. If there is a significantly increased rate of mortality in one of the groups, there will be an early termination of the trial.

### Sample size calculation

The sample size calculation is based on the primary outcome parameter CCI [17] in a non-inferiority design. Assumptions are made on in-house calculations of the CCI on data from the DISPACT trial [24] and the NURIMAS Pancreas study [25] (mean CCI 30; standard deviation 20). A decrease of the CCI by 10 points is considered relevant by patients and clinicians. Therefore, a margin of 7.5 CCI points will be tolerated as non-inferior. With a significance level of 2.5% and 80% power, we need to analyze 113 patients in each group (Nquery 7.0). With an additional dropout of 10%, 126 patients need to be allocated to the no drain and drain arms, respectively. Based on the department’s data, recruitment will be completed within 36 months after inclusion of the first patient.

### Statistical analysis

Non-inferiority of no drain versus drain after distal pancreatectomy will be assessed using a one-sided *t* test. The one-sided significance level is set to 2.5%. The hypotheses to be assessed in the primary efficacy analysis are as follows: H0: μNo Drainage – μDrainage ≥ δ and H1: μNo Drainage – μDrainage < δ, where δ = 7.5 represents the chosen non-inferiority margin and μNo Drainage, μDrainage denote the mean CCI in the no drain and drain groups, respectively. The primary efficacy analysis will be based on the intention-to-treat set according to the intention-to-treat principle. Additionally, an evaluation of the primary outcome will be performed in the per protocol set and in the as treated set (where all patients will be analyzed as they were treated) as sensitivity analyses. Missing data for the primary outcome variable will be replaced by using multiple imputation [26], which takes the covariate treatment group into account by application of the fully conditional specification method. All secondary outcomes will be evaluated descriptively, and descriptive *p* values will be reported together with 95% confidence intervals for the corresponding effects. All analyses will be done using program R 3.2 or higher. Statistical analysis will be performed after closure of the database.

### Methods for minimizing bias

#### Minimizing selection bias

All patients will be consecutively screened and, if found to be eligible, will provide informed consent. The number of screened, included, and analyzed patients will be reported and differences will be explained. The patient flow and the Consolidated Standards of Reporting Trials (CONSORT) flowchart will be reported in the final analysis.

#### Minimizing attrition bias

Statistical measurements such as imputation will be taken to minimize risk of bias due to incomplete outcome data. The trial is registered with the German Clinical Trials Register (Deutsches Register Klinischer Studien), DRKS00013763. To avoid the risk of selective reporting, the trial protocol with full information about endpoints and a profound explanation of planned statistical analysis is hereby published according to the Standard Protocol Items: Recommendations for Interventional Trials (SPIRIT) statement where appropriate [27]. The SPIRIT checklist is provided as Additional file 1.

## Discussion

Distal pancreatectomy for pancreatic diseases is the second most common surgical procedure on the pancreas. Despite the increasing caseload, the morbidity associated with this kind of operation remains high, even in specialized centers [7, 8]. Additional diagnostic and therapeutic procedures are needed for these patients, leading to prolongation of their hospital stay, readmission, and an increase in treatment costs [28]. Several randomized and non-randomized trials have attempted to identify risk constellations where placement of abdominal drains after pancreatic surgery is justified [7, 10–13]. Other studies have tried to explore the effectiveness of early versus late removal of abdominal drains [14, 15].

In 2001, Conlon et al. [29] published the first single-center randomized trial on the usefulness of drains in pancreatic surgery. This study included 139 patients with pancreatoduodenectomy and 40 patients with distal pancreatectomy, with 88 patients randomized to drain and 91 to no-drain. Conlon et al. could show that there was no difference in morbidity, reintervention, or mortality rate. Therefore, these authors concluded that drains should not be considered mandatory or standard after pancreatic resection [29]. In 2011, Van Buren et al. [30] set up a similar multicenter randomized prospective trial to investigate the usefulness of an abdominal drain in patients undergoing pancreatoduodenectomy as well as distal pancreatectomy. However, in 2014 this trial had to be terminated prematurely after 137 patients by a Data Safety Monitoring Board decision because of a higher mortality frequency in the no-drain group in patients with pancreatoduodenectomy. In contrast to Conlon et al., these researchers could show decreased mortality and morbidity with routine drainage after pancreatoduodenectomy [30]. In 2015, Dou et al. [31] published a systematic review and meta-analysis of prophylactic abdominal drainage after pancreatic resection to investigate whether prophylactic abdominal drainage is necessary after pancreatic resection or not. They included nine eligible studies involving a total of 2794 patients in this meta-analysis and showed that placement of prophylactic drainage did not have beneficial effects on clinical outcomes, including morbidity, postoperative pancreatic fistula, reoperation, interventional radiology drainage, and length of hospital stay. Additionally, prophylactic drainage did not significantly increase the risk of abdominal abscess. Omitting prophylactic abdominal drainage resulted in higher mortality after pancreatectomy. Therefore, Dou et al. still recommended prophylactic abdominal drainage after pancreatic resection [31]. In 2016, Witzigmann et al. could show that the omission of drains in patients with pancreatic head resection and pancreatic anastomosis was not inferior to intra-abdominal drainage in terms of postoperative reintervention and superior in terms of clinically relevant pancreatic fistula rate and fistula-associated complications [16]. According to Witzigmann et al., there seems to be no need for routine prophylactic drainage after pancreatic resection with pancreaticojejunal anastomosis. In 2017, Van Buren et al. [32] published another multicenter trial including a total of 344 patients who underwent distal pancreatectomy with and without the use of intraperitoneal drainage (174 versus 170 patients). In contrast to the results of 2014, Van Buren et al. showed that there was no difference in the rate of grade II or higher complications and also no difference in clinically relevant postoperative pancreatic fistula or mortality. However, distal pancreatectomy without routine intraperitoneal drainage was associated with a higher incidence of intra-abdominal fluid collection. Nevertheless, there was no difference in the frequency of postoperative imaging, percutaneous drain placement, reoperation, readmission, or quality of life scores [32]. In 2017, Huettner et al. published a meta-analysis of studies comparing abdominal drainage with no drainage after pancreatic surgery. They included the randomized controlled trials of Conlon et al. [29], Van Buren et al. [30], and Witzigmann et al. [32] and showed that pancreatic resection with or without abdominal drainage resulted in similar rates of mortality, morbidity, and reintervention [33].

Some surgeons have abandoned the routine use of drains in pancreatic surgery, but many surgeons still fear that abandoning prophylactic drainage after a pancreatic resection may be hazardous and result in an increase of potentially severe complications. On the other hand, the possibility of drain-related morbidity needs to be considered, and routine drainage should not be accepted as a historic standard without being allowed to challenge this. As the above-mentioned studies on drainage practice in pancreatic surgery show conflicting results, the scientific discussion may not be terminated after completion of one randomized controlled trial for a specific topic. The ongoing intense debate on whether routine abdominal drainage after distal pancreatectomy has any effect on the reduction of postoperative complications shows the need for more high-quality studies. Consequently, the present single-center PANDRA II trial will provide additional evidence for the question of routine drain placement in distal pancreatectomy and will increase the available evidence in this field in addition to the already-published studies. The aim of the present single-center RCT is to evaluate the outcome after open or minimally invasive pancreatic distal pancreatectomies concerning the presence or absence of prophylactic abdominal cavity drainage. Specific outcome endpoints are postoperative complications.

### Trial status

The PANDRA II trial recruitment started in April 2018 and is currently ongoing. The last patient is expected to be recruited early in 2021.

## Additional files

Additional file 1: SPIRIT 2013 checklist: recommended items to address in a clinical trial protocol and related documents. (DOC 121 kb)

Additional file 2: Classification of surgical complications according to Clavien-Dindo [18]. (DOCX 14 kb)

## Data Availability

Not applicable.

## Abbreviations

CCI: Comprehensive Complication Index
CT: Computed tomography
DOD: Day of discharge
IMBI: Institute of Medical Biometry and Informatics, University of Heidelberg, Heidelberg, Germany
ISGPS: International Study Group of Pancreatic Surgery
KSC: Clinical Trial Center, University of Heidelberg, Heidelberg, Germany
POD: Postoperative day
