# Supplementary material for: Abdominal drainage versus no drainage after distal pancreatectomy: study protocol for a randomized controlled trial
Source: Trials. 2019 Jun 7;20:332. doi: 10.1186/s13063-019-3442-0 (PMC6555976; doi:10.1186/s13063-019-3442-0)
Supplement: Supplementary file 2 — Classification of surgical complications according to Clavien-Dindo [18]. (DOCX 14 kb) [file 13063_2019_3442_MOESM2_ESM.docx]

**Supplement**

**Classification of Surgical Complications according to Clavien-Dindo Classification [18]**

|  | |
| --- | --- |
| Grade I | Any deviation from the normal postoperative course without the need for pharmacological treatment or surgical, endoscopic, and radiological interventions. Allowed therapeutic regimens are: drugs as antiemetics, antipyretics, analgetics, diuretics, electrolytes, and physiotherapy. This grade also includes wound infections opened at the bedside. |
| Grade II | Requiring pharmacological treatment with drugs other than such allowed for grade I complications Blood transfusions and total parenteral nutrition are also included. |
| Grade III | Requiring surgical, endoscopic or radiologicalintervention. |
| Grade IIIa | Intervention not under general anesthesia. |
| Grade IIIb | Intervention under general anesthesia. |
| Grade IV | Life-threatening complication (including CNS complications)* requiring IC/ICU management. |
| Grade IVa | Single organ dysfunction (including dialysis). |
| Grade IVb | Multiorgan dysfunction. |
| Grade V | Death of a patient. |
| Suffix “d” | If the patient suffers from a complication at the time of discharge, the suffix “d” (for “disability”) is added to the respective grade of complication. This label indicates the need for a follow-up to fully evaluate the complication. |
| *Brain hemorrhage, ischemic stroke, subarachnoidal bleeding, but excluding transient ischemic attacks. CNS, central nervous system; IC, intermediate care; ICU, intensive care unit. | |
|  | |
